# Basal and dynamics mRNA expression of muscular HSP108, HSP90, HSF-1 and HSF-2 in thermally manipulated broilers during embryogenesis

**DOI:** 10.1186/s12917-019-1827-7

**Published:** 2019-03-08

**Authors:** Mohamed Borhan Al-Zghoul, Sabry Mohamed El-Bahr

**Affiliations:** 1 Department of Basic Medical Veterinary Sciences, Faculty of Veterinary Medicine, Jordan University of Science and Technology, P.O. Box 3030, Irbid, 22110 Jordan; 2 Department of Physiology, Biochemistry and Pharmacology, College of Veterinary Medicine, King Faisal University, P.O. Box 400, Al-Hufof, 31982 Saudi Arabia; 3 Department of Biochemistry, Faculty of Veterinary Medicine, Alexandria University, Alexandria, Egypt

**Keywords:** Heat shock protein (HSP), Heat shock factor (HSF), Thermotolerance, Thermal manipulation (TM)

## Abstract

**Background:**

Limited data are available about the kinetics of mRNA expressions of Heat shock proteins (HSPs) and heat shock factors (HSFs) in the thermally manipulated (TM) broiler chicks during acute heat stress. Therefore, this study aimed to investigate effects of thermal manipulation (TM) of broiler chicken during embryonic days (ED) 12–18 on the basal and dynamics mRNA expression of heat shock proteins (HSP108 and HSP90) and heat shock factors (HSF-1 and HSF-2) in the muscle tissue during late embryogenesis, first week of life and during heat stress (HS) on post-hatch days 14 and 28. One thousand and five hundred fertile Ross 315 broiler eggs were randomly allocated to five groups: control group (37.8 °C), TM_1_ (38.5 °C for 18 h), TM_2_ (39 °C for 18 h), TM_3_ (39.5 °C for 18 h) and TM_4_ (40 °C for 18 h). Chicks from each treatment group were then randomly sub-divided into two further treatment groups, naïve and thermal challenged (TC). On post-hatch days 14 and 28, thirty chicks from each TC group were subjected to heat stress (41 °C for 6 h), while naïve chicks of each group (*n* = 30) were kept under regular conditions. The response of chicks to heat stress was investigated by evaluating the change in mRNA expressions of HSP108, HSP90, HSF-1 and HSF-2 in muscle tissue after 1, 3 and 5 h of heat stress.

**Results:**

When compared to the control group, TM resulted in significant increases in the basal mRNA expression of HSPs and HSFs during embryogenesis and altered their dynamic expressions in the muscle tissue after heat stress on post-hatch days 14 and 28.

**Conclusion:**

the current study indicated short- and long-term enhancement of HSPs and HSFs gene expression which was associated with acquisition of improved thermotolerance in thermally manipulated chicks.

## Background

High temperature has adverse impact on the broiler chicken’s physiology, biochemistry and growth performance with a negative effect on poultry industry [1]. The higher sensitivity of broiler chickens to high ambient temperatures compare to other species of domestic animals attributed to their higher body temperature, rapid metabolism and absence of sweat glands as well [2]. Acute heat stress reduced the growth performance and meat quality and increased the morbidity and mortality rates with subsequent economic losses in in broilers industry [3, 4]. Under heat stress conditions, genes related to cell survival and stabilities are upregulated, while less vital genes are downregulated [5]. Thermal stressors stimulate animal tissues for rapid synthesis of highly conserved proteins known as heat shock proteins [5, 6]. From biochemical point of view, HSPs maintain protein integrity by avoiding protein aggregation refolding of damaged proteins [6, 7]. Based on molecular size, HSPs classified into main six families, namely HSP100, HSP90, HSP70, HSP60, HSP40, and the small HSPs [8–11]. Chicks who were subjected to thermal manipulation during particular periods of embryogenesis were able to reduce their heat production during heat stress later in their life by regulating the thyroid metabolism [12–18] and/or stimulating of heat shock proteins biosynthesis [19]. Previously, we reported that, different TM protocols during broiler chicken embryogenesis resulted in significant alterations in the mRNA expressions of HSPs (HSP108, HSP90, HSP70, HSP60 and HSP47) and HSFs (HSF-1, HSF-3 and HSF-4), which were associated with improve thermotolerance acquisition during heat stress later in the broiler chickens’ life [19–23]. Our recent published work [19] indicated for the first time that, similar TM protocols (TM_1_:38.5 °C; TM_2_ 39 °C; TM_3_: 39.5 °C; TM_4_: 40 °C for 18 h) increased basal mRNA levels and altered production dynamics of HSP70, HSP60 and HSF-3 and HSF-4 during thermal stress. The current study is a continuation of our recent published work [19] and aimed to establish a multi-time-point evaluation of the basal and dynamics mRNA expression of HSP108, HSP90, HSF-1 and HSF-2 at embryonic life (ED 12 and 18), post-hatch days 1, 5 and 7 and during heat stress at post-hatch days 14 and 28 in muscle of broilers chickens exposed to different TM protocols (TM_1_:38.5 °C; TM_2_ 39 °C; TM_3_: 39.5 °C; TM_4_: 40 °C for 18 h) during embryogenesis (ED 12–18).

## Methods

### Incubating and hatching management

The experimental procedures and management protocols used in this study were carried out in accordance with the national institute of health guidelines for the care and the use (NIH Publications No.8023, revised 1978) and approved by the Jordan University of Science and technology Animal Care and Use Committee (JUST-ACUC; permission #16/3/3/390). A total of 1700 fertile Ross 315 broiler eggs were purchased from certified Ross breeder flock of hens (Irbid, Jordan). A total of 1500 normal eggs were selected, for an initial weight 64 ± 2 g and incubated in five semi-commercial incubators (types 25 HS-SSF, Masalles, Barcelona, Spain). The selected eggs divided into five incubation treatment groups (300 each): control group was maintained at 37.8 °C 56% relative humidity (RH) throughout the incubation period; TM_1_ was subjected daily to TM at 38.5 °C for 18 h and 65% RH during ED12–18; TM_2_ was subjected daily to TM at 39 °C for 18 h and 65% RH during ED12–18; TM_3_ was subjected daily to TM at 39.5 °C for 18 h and 65% RH during ED12–18 and TM_4_ was subjected daily to TM at 40 °C for 18 h and 65% RH during ED12–18. TM1-TM4 were incubated like the control condition (37.8 °C, 56% RH) during the last 4 h of the day. At hatch, the number of hatched chicks was recorded hourly. The one-day old chicks were transferred to animal house of Jordan University of Science and technology where the field experiment was conducted. Chicks were distributed in cage pens at room temperature 33 °C and the temperature was gradually decreased to 24 °C. Starting from post-hatch day 24 until day 35, the temperature was maintained at 21 °C. Water and feed were provided to the chicks ad libitum.

### Thermal manipulation and heat stress

To evaluate the effect of TM during embryogenesis on thermotolerance acquisition, chicks from each treatment group were randomly divided into two subgroups, naïve (N) and thermal challenge (TC). On post-hatch days 14 and 28, thirty randomly selected chicks from each TC groups were thermally stressed by adjusting room temperature to 41 °C for 6 h. Thirty naïve chicks of each group were kept thermo-neutral condition (25 ± 1 °C and 50–60% RH) in a separated room. Our team [19] recently published the data of body temperature of chicks during post-hatch days (1–35) and after 0, 1, 3 and 5 h from beginning of thermal stress. After 1, 3 and 5 h from the beginning of heat stress, five chicks from each treatment group were humanely euthanatized and samples from pectoral and thigh muscle were collected for total RNA isolation and semi-quantitative real time RT-PCR analyses. Euthanasia was performed after sodium pentobarbital anesthesia (20–30 mg/kg; [24]). Sodium pentobarbital was injected to radial vein with sterilized needles. The same samplings and measurements protocols were conducted using the naïve chicks as controls.

### Total RNA extraction and reverse transcription

Muscle mRNA expression of HSPs and HSFs were evaluated using the semi-quantitative real time RT-PCR analyses. Pectoral and thigh samples were collected at embryonic life (ED 12 and 18), post-hatch days 1, 5 and 7 and during heat stress at post-hatch days 14 and 28. Muscles samples were collected from 50 embryos at ED 12 and 18 (5 embryo from each treatment group per day), from 75 chicks (5 chicks from each treatment group per day) at post-hatch days 1, 5 and 7 and from 200 chicks (5 chicks from each treatment group per time point per day; 5 chicks×5groups × 4 time points× 2 days) at post-hatch days 14 and 28. Total RNAs isolation, concentration, reverse transcription and cDNA synthesis were performed as described earlier [19].

### Semi-quantitative real-time RT-PCR

Semi-Quantitative real-time RT-PCR was performed using QuantiFast SYBR® Green PCR Kit (Qiagen, Valencia, CA, USA) on a Rotor-Gene Q Real-Time PCR system (Qiagen, Valencia, CA, USA) as described earlier [19].

### Primers

The following primer sequences were used in the real-time RT-PCR analyses: **(cGAPDH):** F-5’GTGTTATCATCTCAGCTCCCTCAG’3, R-5’GGTCATAAGACCCT CCACAATG3’; **(cHSP108)**: F-5’ATGTGTGGAGCAGCAAGACAGAGA’3, R-5’TTC ATGAGCTCCCAATCCCAGACA’3; (**cHSP90):** F-5’ACTCTGCTTACCTTGTTGCGG AGA’3, R-5’TCCTTGTTCGCCGTTCTTCCAGA’3; (**cHSF-1):** F-5’TCCATGTGTTCGA CCAAGGACAGT’3, R-5’TGGAACTCAGTGTCGTCCTTCTCT’3; **(cHSF-2):** F-5’CCA GCTGCTTCACAGGAAACACAA, R-5’AG AGGAAGGAGTTTCAGTTGCGGA.

### Statistical analysis

All statistical analyses were performed using IBM SPSS statistics 23 software (IBM software, Chicago, USA). Data for the HSPs and HSFs were expressed in means±SD. For each experiment time point (ED 12 and 18, post-hatch days 1, 5, 7, 14 and 28), One-way analysis of variance (ANOVA), followed by an all-pairs Bonferroni test, was used to compare different parameters in all treatment groups (control vs. TM groups) and two-way analysis of variance (ANOVA) was used to compare mRNA fold changes within the same groups (naïve (0 h) vs. 1, 3 and 5 h of heat stress). The mRNA fold change in the expression was considered to be significant if the *P* values obtained were less than 0.05 (*P* < 0.05).

## Results

### Effects of TM on the mRNA expression of HSP108, HSP90, HSF-1 and HSF-2 in the muscle at ED 12 and 18

**Fig. 1:**
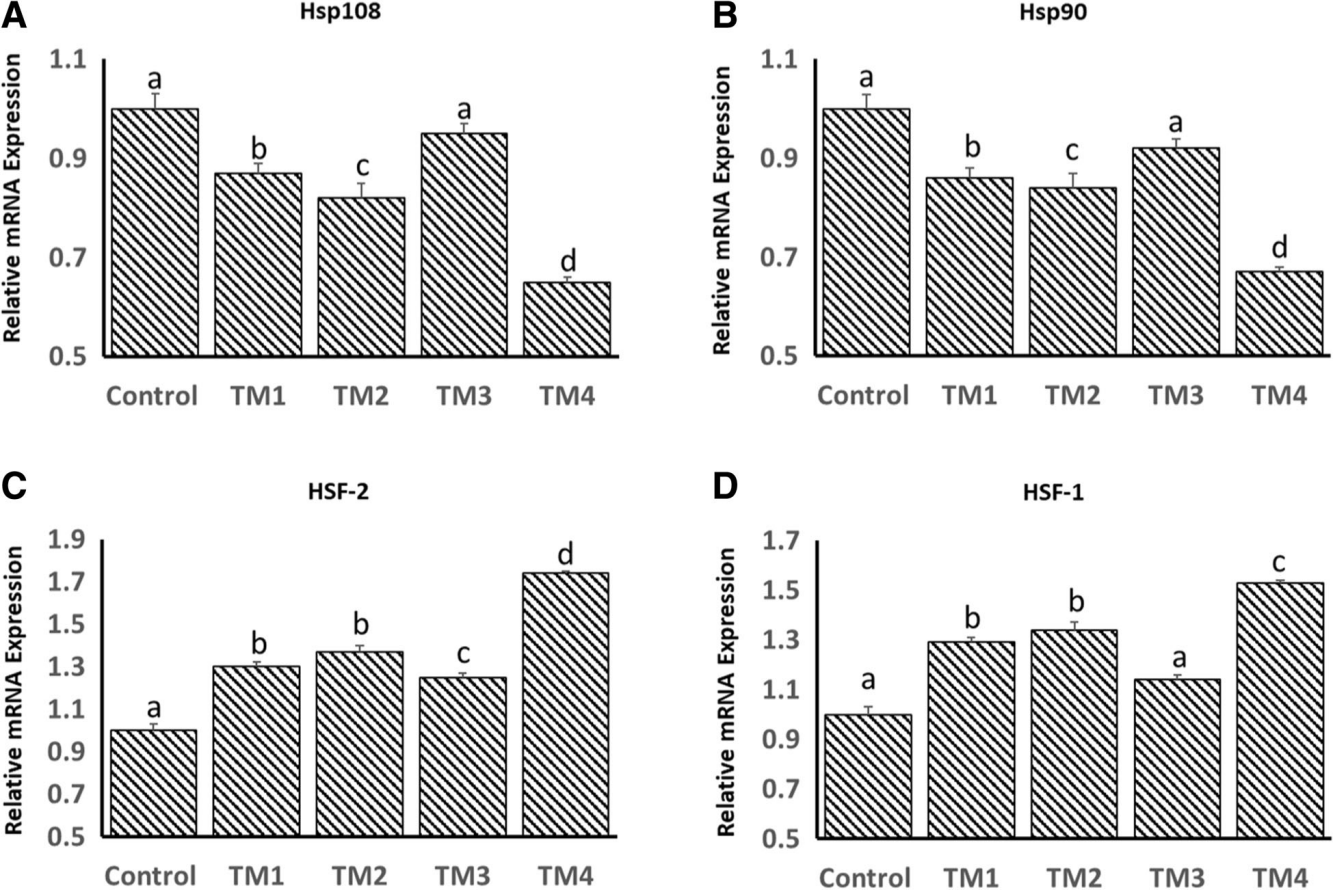
Effect of different thermal manipulation protocols (TM1, TM2, TM3 and TM4) daily during the embryonic days (ED) 12–18 on the mRNA expressions of **a**) Hsp108, **b**) Hsp90, **c**) HSF-1 and **d**) HSF-2 at ED 12. Control = 37.8°C; TM_1_ = Thermal manipulation at 38.5°C daily for 18 h; TM_2_ = Thermal manipulation at 39 °C daily for 18 h; TM_3_ = Thermal manipulation at 39.5°C daily for 18 h and TM_4_ = Thermal manipulation at 40 °C daily for 18 h. ^a–d^ Within the same gene, means ± SD with different superscripts differ significantly (*p* < 0.05)

**Fig. 2:**
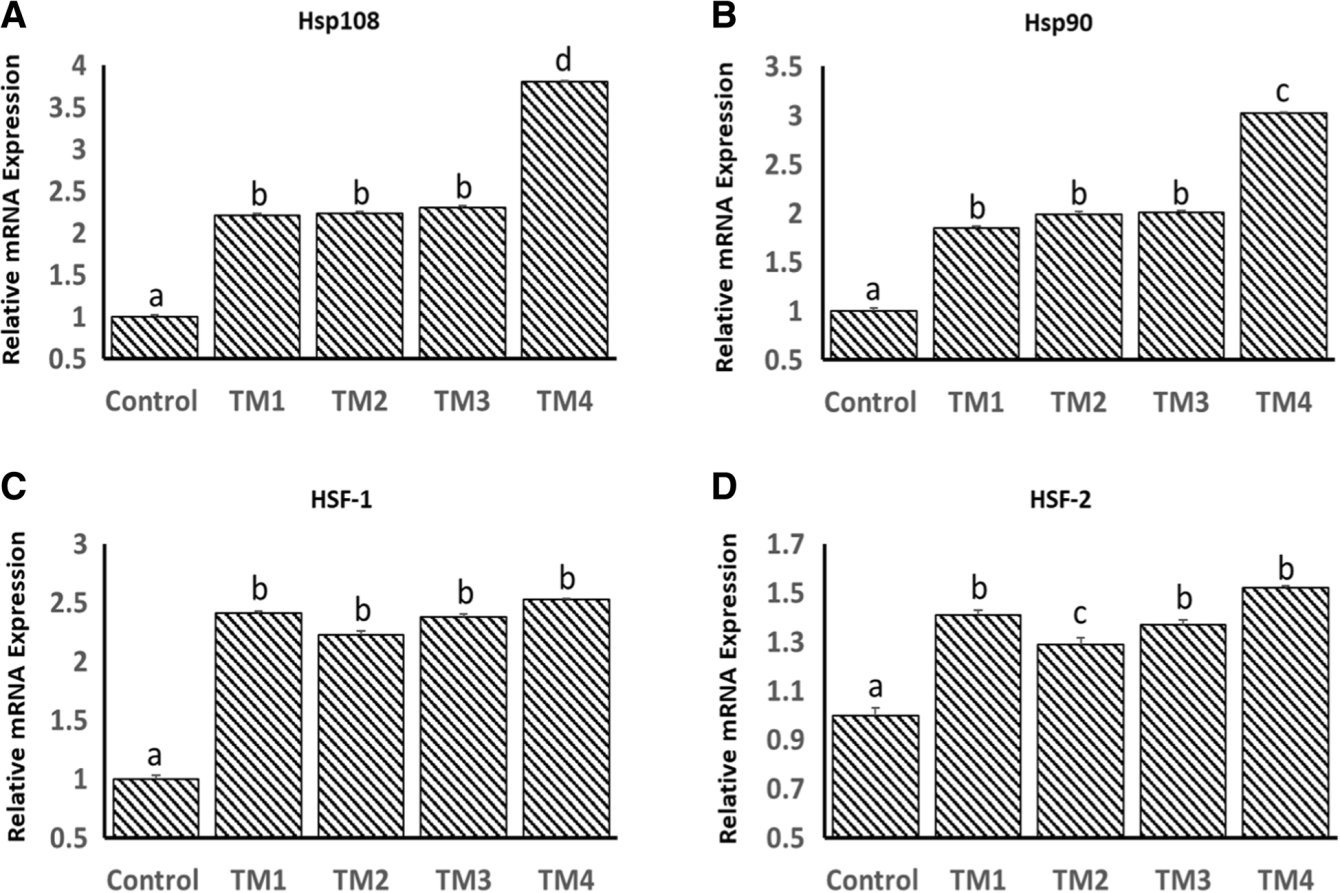
Effect of different thermal manipulation protocols (TM1: TM2: TM3 and TM4) daily during the embryonic days (ED) 12–18 on the mRNA expressions of **a**) Hsp108, **b**) Hsp90, **c**) HSF-1 and **d**) HSF-2 at ED 18. Control = 37.8°C; TM_1_ = Thermal manipulation at 38.5°C daily for 18 h; TM_2_ = Thermal manipulation at 39 °C daily for 18 h; TM_3_ = Thermal manipulation at 39.5°C daily for 18 h and TM_4_ = Thermal manipulation at 40 °C daily for 18 h. a–d Within the same gene, means ± SD with different superscripts differ significantly (*p* < 0.05)

As indicated in our recently published work [19], TM treatments had no effect on the body temperature at any stage of development (post-hatch days 1–35). However, during heat stress on post-hatch days 14 and 28, the body temperature of thermally challenged chicks was significantly lower than that of the controls (for details see [19]). Effects of TM on the mRNA expression of HSP108, HSP90, HSF-1 and HSF-2 in the muscle at ED 12 and 18 are shown in Figs. 1 and 2, respectively. At ED 12, mRNA expression of HSP108 and HSP90 was significantly lower in all TM groups, except TM3, compared to the controls (Fig. 1a and b). In contrast, TM induced significant increases in mRNA expression of HSF-1 in all TM groups except for TM3 compared to the controls (Fig. 1d). Moreover, TM induced significant increases in mRNA expression of HSF-2 in all TM groups compared to the controls (Fig. 1c). AT ED 18, mRNA expression of HSP108, HSP90, HSF-1 and HSF-2 was significantly higher in all TM groups compared to the control groups (Fig. 2a-d).

### Effects of TM on the basal mRNA expression of HSP108, HSP90, HSF-1 and HSF-2 on post-hatch days 1, 5 and 7

**Fig. 3:**
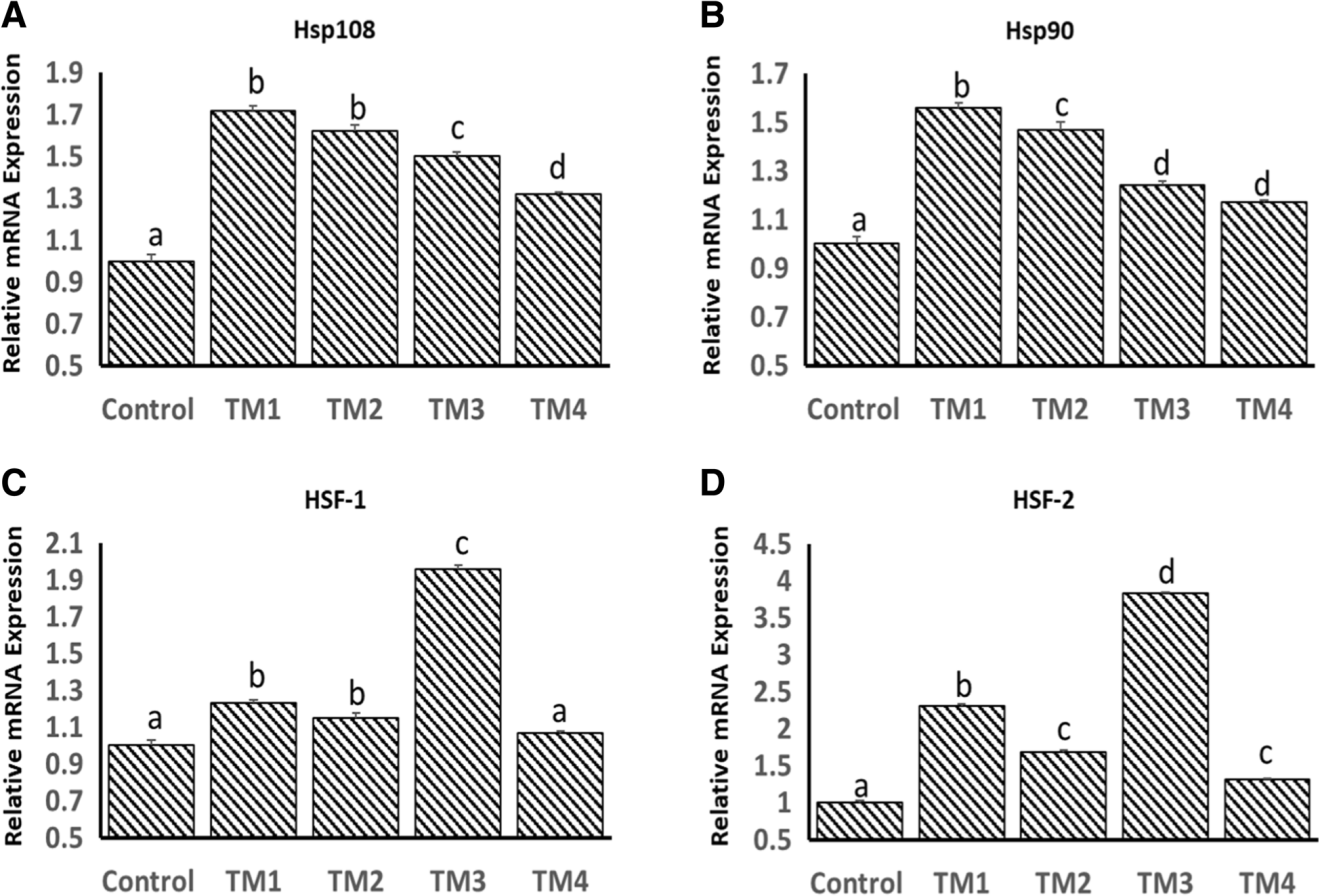
Effect of different thermal manipulation protocols (TM1: TM2: TM3 and TM4) daily during the embryonic days (ED) 12–18 on the mRNA expressions of **a**) Hsp108, **b**) Hsp90, **c**) HSF-1 and **d**) HSF-2 on post-hatch day 1. Control = 37.8°C; TM_1_ = Thermal manipulation at 38.5°C daily for 18 h; TM_2_ = Thermal manipulation at 39 °C daily for 18 h; TM_3_ = Thermal manipulation at 39.5°C daily for 18 h and TM_4_ = Thermal manipulation at 40 °C daily for 18 h. a–d Within the same gene, means ± SD with different superscripts differ significantly (*p* < 0.05)

**Fig. 4:**
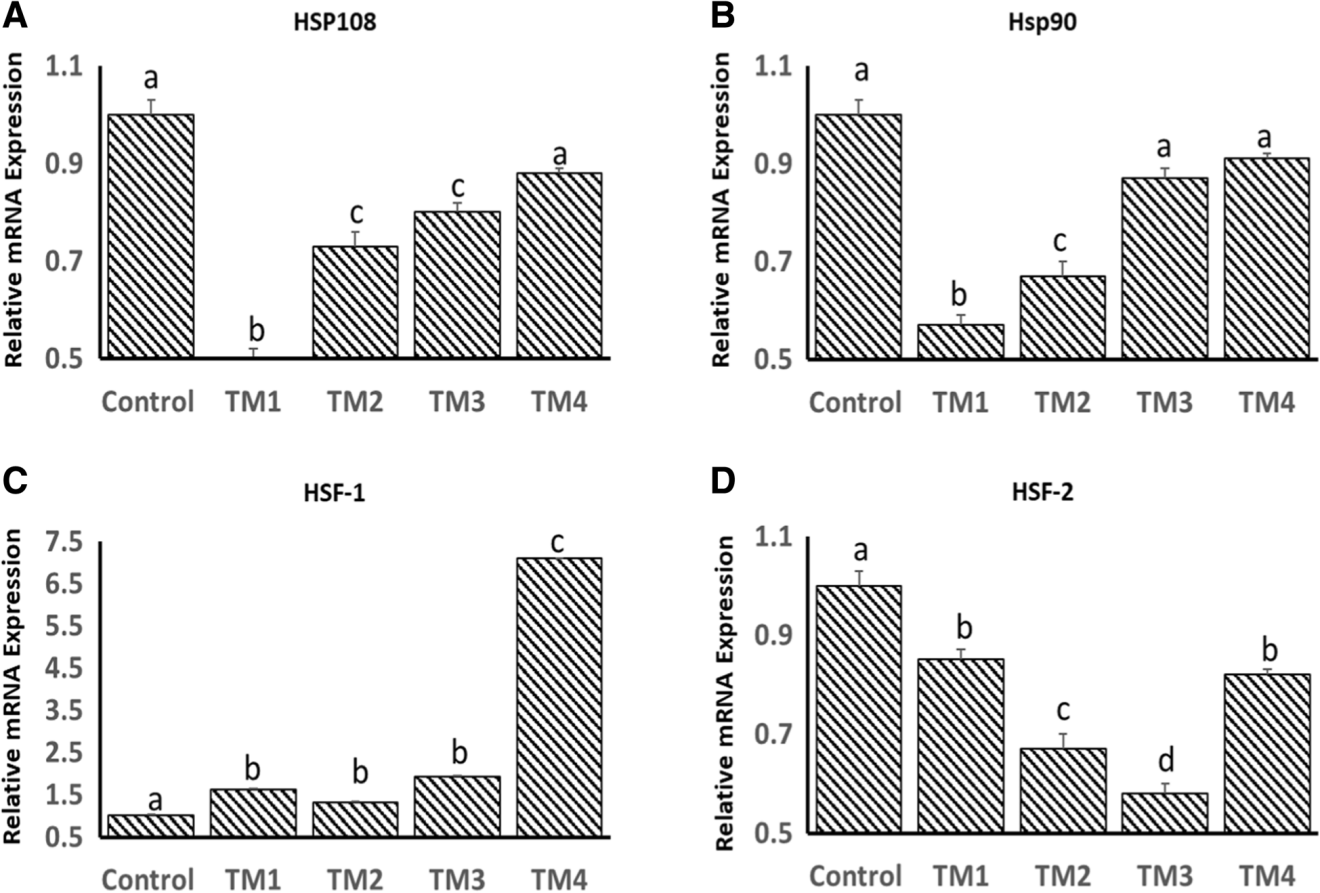
Effect of different thermal manipulation protocols (TM1: TM2: TM3 and TM4) daily during the embryonic days (ED) 12–18 on the mRNA expressions of **a**) Hsp108, **b**) Hsp90, **c**) HSF-1 and **d**) HSF-2 on post-hatch day 5. Control = 37.8°C; TM_1_ = Thermal manipulation at 38.5°C daily for 18 h; TM_2_ = Thermal manipulation at 39 °C daily for 18 h; TM_3_ = Thermal manipulation at 39.5°C daily for 18 h and TM_4_ = Thermal manipulation at 40 °C daily for 18 h. a–d Within the same gene, means ± SD with different superscripts differ significantly (*p* < 0.05)

**Fig. 5:**
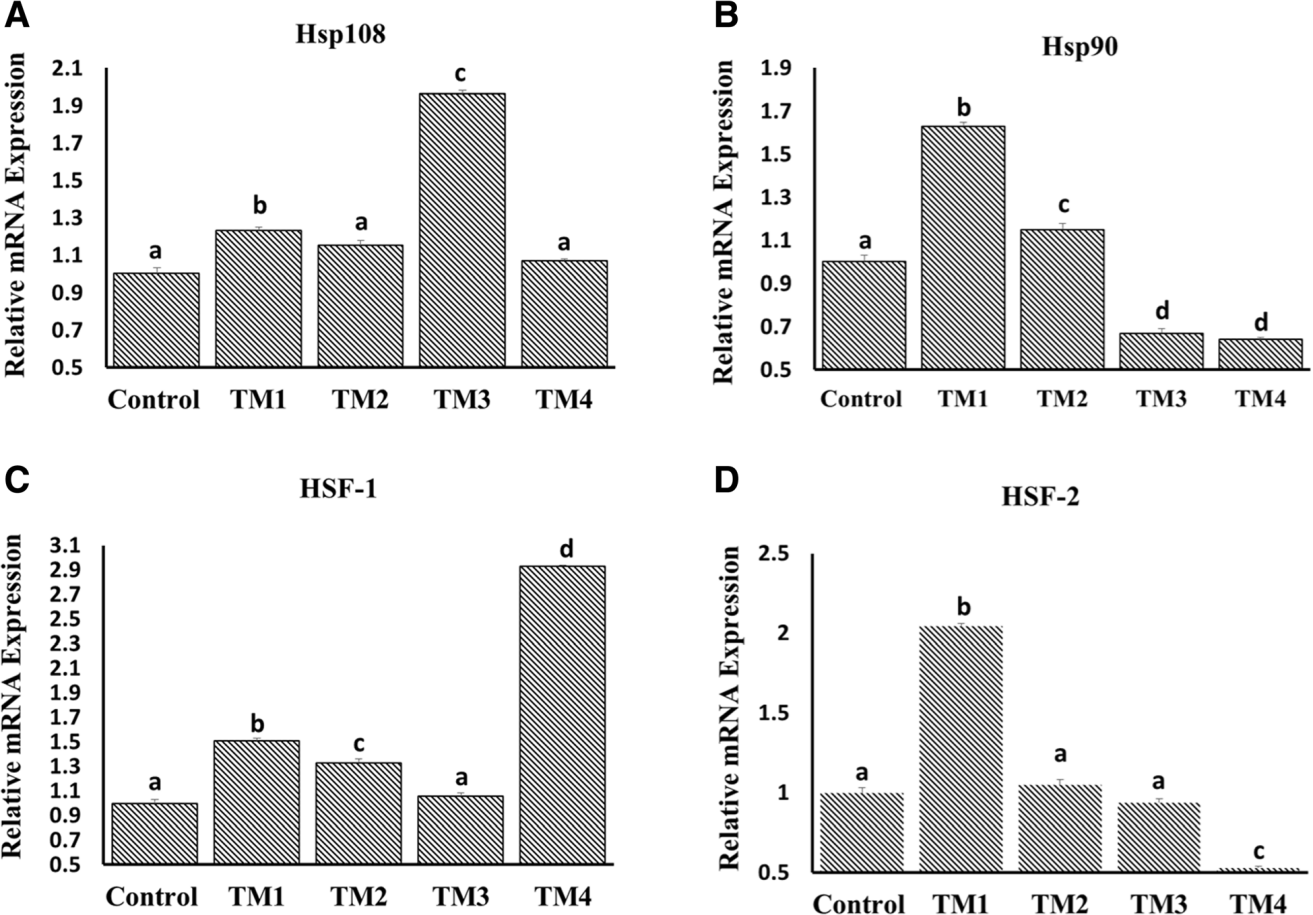
Effect of different thermal manipulation protocols (TM1: TM2: TM3 and TM4) daily during the embryonic days (ED) 12–18 on the mRNA expressions of **a**) Hsp108, **b**) Hsp90, **c**) HSF-1 and **d**) HSF-2 on post-hatch day 7. Control = 37.8°C; TM_1_ = Thermal manipulation at 38.5°C daily for 18 h; TM_2_ = Thermal manipulation at 39 °C daily for 18 h; TM_3_ = Thermal manipulation at 39.5°C daily for 18 h and TM_4_ = Thermal manipulation at 40 °C daily for 18 h. a–d Within the same gene, means ± SD with different superscripts differ significantly (*p* < 0.05)

Effects of TM on the basal mRNA expression of HSP108, HSP90, HSF-1 and HSF-2 in the muscle on post-hatch days 1, 5 and 7 are shown in Figs. 3, 4 and 5. On post-hatch day 1, mRNA expression of HSP108, HSP90 and HSF-2 was significantly higher in all TM groups compared to the controls (Fig. 3a, b and d). On post-hatch day 1, mRNA expression of HSF-1 was significantly higher in all TM groups except for TM4 compared to the controls (Fig. 3c). On post-hatch day 5, mRNA expression of HSP108 was significantly lower in all TM groups except for TM4, which remained comparable to the controls (Fig. 4a). On post-hatch day 5, mRNA expression of HSP90 was significantly lower in TM1 and TM2 groups compared to TM3 and TM4, which remained comparable to the controls (Fig. b). On post-hatch day 5, mRNA expression of HSF-2 was significantly lower in all TM groups compared to the controls (Fig. 4d). In the other hand, significant increases in mRNA expression of HSF-1 in all TM groups compared to the controls were found (Fig. 4c). On post-hatch day 7, TM resulted in significant increases in mRNA expression of HSP108 in TM_1_ and TM_3_ groups compared to TM_2_, TM_4_ and control groups (Fig. 5a). In contrast, except for significant increases in mRNA expression of HSP90 in the TM_1_ and TM_2_ groups, TM resulted in a significant reduction in mRNA expression of HSP90 in TM_3_ and TM_4_ (Fig. 5b). TM resulted in significant increases in mRNA expression of HSF-1 in TM_1_, TM_2_ and TM4 compared to TM_3_ and control groups (Fig. 5c). Furthermore, except for a significant increase in mRNA expression of HSF-2 in TM1 and a significant reduction in TM_4_, comparable mRNA levels of HSF-2 were observed in control, TM_2_ and TM_3_ groups (Fig. 5d).

### Effect of TM and heat stress (41 °C for 6 h) on mRNA expression of HSPs and HSFs in the muscle tissue on post-hatch day 14

#### mRNA expression of HSPs

Effects of TM and heat stress on mRNA expression of HSPs in muscle tissue on post-hatch day 14 are shown in Table 1. Before heat stress (0 h, naïve), the basal mRNA expression of HSP108 in TM groups was significantly higher than in the control group. Furthermore, after 1 h of heat stress, significant increases in mRNA expression of HSP108 were observed in all treatment groups and levels remained higher after 3 h of heat stress. However, after 5 h of heat stress, a decline in mRNA expression of HSP108 was observed in all TM groups compared to the basal expression (0 h). The peak of RNA expression of HSP108 in TM_1_, TM_2_, TM_3_ and control groups was observed 3 h after the beginning of heat stress, whereas in TM_4_, the peak was observed 1 h after the beginning of heat stress. Before heat stress (0 h, naïve), the basal mRNA expression of HSP90 in all TM groups was significantly higher compared to the control group. Furthermore, after 1 and 3 h of heat stress, significant increases in the mRNA expression of HSP90 in all treatment groups were observed and these remained after 3 h of heat stress. However, at 5 h after the start of heat stress, a decline in mRNA expression of HSP90 was observed in TM1 and TM4 groups compared to TM2 and TM3 that remained comparable to the basal expression (0 h). Peak mRNA expression of HSP90 in TM_1_, TM_2_, TM_3_ and control groups was observed 3 h after the beginning of heat stress, whereas for the TM4 group, the peak of mRNA level of HSP90 was observed after 1 h of heat stress.

**Table 1:** The effect of TC (41 °C for 6 h; Post- Hatch Day 14) during 4 time terminals (at 0, 1, 3 and 5 h of TC) on relative mRNA expression of Hsp108, Hsp90, HSF-1 and HSF-2 in the Muscle of broiler chicks subjected to different TM protocols at ED 12–18 (*n* = 5)

Parameters	Groups	Treatments				
		Control	TM_1_	TM_2_	TM_3_	TM_4_
Hsp108	TN	1.00 ± 0.02^aw^	2.96 ± 0.03^bw^	1.63 ± 0.01^cw^	1.53 ± 0.01^cw^	2.28 ± 0.03^dw^
	TC 1	4.87 ± 0.02^ax^	5.09 ± 0.08^bx^	6.37 ± 0.07^cx^	3.48 ± 0.08^dx^	11.9 ± 0.27^ex^
	TC 3	6.86 ± 0.12^ay^	8.98 ± 0.06^by^	6.80 ± 0.06 ^ay^	8.33 ± 0.04^by^	6.22 ± 0.01^cy^
	TC 5	1.40 ± 0.03^az^	1.47 ± 0.05^az^	1.58 ± 0.06^bw^	1.24 ± 0.07^cz^	1.82 ± 0.04^dz^
Hsp90	TN	1.00 ± 0.01^aw^	2.90 ± 0.01^bw^	1.57 ± 0.02^cw^	1.35 ± 0.02^dw^	2.32 ± 0.03^ew^
	TC 1	5.10 ± 0.03^ax^	4.74 ± 0.06^bx^	6.15 ± 0.06^cx^	3.22 ± 0.06^dx^	12.1 ± 0.29^ex^
	TC 3	6.96 ± 0.32^ay^	8.54 ± 0.05^by^	6.54 ± 0.16^ax^	7.55 ± 0.04^cy^	6.06 ± 0.07^dy^
	TC 5	1.50 ± 0.06^az^	1.42 ± 0.04^az^	1.80 ± 0.01^bw^	1.35 ± 0.06^aw^	1.85 ± 0.05^bz^
HSF-1	TN	1.00 ± 0.02^aw^	1.07 ± 0.02^aw^	0.99 ± 0.01^aw^	1.36 ± 0.05^bw^	0.98 ± 0.02^aw^
	TC 1	0.56 ± 0.08^ax^	0.40 ± 0.07^ax^	1.61 ± 0.05^bx^	0.85 ± 0.04^cx^	2.84 ± 0.06^dx^
	TC 3	0.98 ± 0.02^aw^	0.93 ± 0.06^ay^	1.21 ± 0.05^by^	0.99 ± 0.03^cx^	0.95 ± 0.04^cw^
	TC 5	0.79 ± 0.01^az^	1.47 ± 0.05^bz^	1.01 ± 0.04^cw^	0.97 ± 0.02^cx^	1.28 ± 0.04^dy^
HSF-2	TN	1.00 ± 0.04^aw^	3.13 ± 0.07^bw^	1.48 ± 0.01^cw^	1.39 ± 0.03^cw^	2.09 ± 0.04^dw^
	TC 1	1.65 ± 0.04^ax^	1.21 ± 0.04^bx^	2.12 ± 0.06^cx^	1.26 ± 0.08^bw^	3.02 ± 0.05^dx^
	TC 3	1.55 ± 0.06^ax^	2.56 ± 0.08^by^	1.56 ± 0.07^aw^	1.71 ± 0.02^cx^	0.75 ± 0.02^dy^
	TC 5	0.29 ± 0.01^ay^	1.97 ± 0.06^bz^	2.41 ± 0.09^cy^	2.48 ± 0.08^cy^	1.67 ± 0.05^dz^

*TC* Thermal challenged, *TM* thermal manipulation, *TN* Naїve (thermal-neutral). Control = 37.8°C; TM_1_ = Thermal manipulation at 38.5°C for 18 h; TM_2_ = Thermal manipulation at 39 °C for 18 h; TM_3_ = Thermal manipulation at 39.5°C for 18 h; TM_4_ = Thermal manipulation at 40 °C for 18 h

^a–e^within rows, means ± SD with different superscripts differ significantly (*P* < 0.05)

^w-z^Between naïve and TC chicks within a parameter, means ± SD with different superscripts differ significantly (*P* < 0.05)

#### mRNA expression of HSFs

Effects of TM and heat stress on the mRNA expression of HSFs in the muscle on post-hatch day 14 are shown in Table 1. Before heat stress (0 h, naïve), the basal mRNA expression of HSF-1 in TM3 group was significantly higher than those in other TM and control groups. However, after 1 h of heat stress, significant increases in mRNA expression of HSF-1 were found in TM2 and TM4, the highest increase occurring in the TM_4_ group. On the other hand, a significant reduction in mRNA expression of HSF-1 was observed in TM_1_, TM_3_ and control groups compared to (0 h, naïve group). However, 3 h after the beginning of heat stress, except for a significant increase in mRNA expression of HSF-1 in the TM_2_ group, comparable mRNA expression of HSF-1 was observed among the treatment groups. Furthermore, after 5 h of heat stress, except for a significant increase in mRNA expression of HSF-1 in the TM1 and TM4 groups, comparable expression of HSF-1 was observed among the treatment groups. Peak mRNA expression of HSF-1 was observed at 1 h after heat stress in TM_2_ and TM_4_ groups, whereas in TM1, a peak was observed at 5 h of heat stress. Prior to heat stress (0 h, naïve), the basal mRNA expression of HSF-2 in all TM groups was significantly higher compared to the control group. However, after 1 h of heat stress, significant increases in mRNA expression of HSF-2 were observed in TM_2_, TM_4_ and control groups compared to those in TM_1_, TM_3_ and. Furthermore, after 3 h of heat stress, a higher level of mRNA expression of HSF-2 was observed in TM_1_ and TM_3_ groups compared to those in TM2, TM4 and control groups. Furthermore, at 5 h of heat stress, a second peak of mRNA expression of HSF-2 occurred in all TM groups compared to the controls.

### **Effect of TM and** heat stress **(41 °C for 6 h) on mRNA expression of HSPs and HSFs in muscle on post-hatch day 28**

#### mRNA expression of HSPs

Effects of TM and heat stress on the mRNA expression of HSPs in the muscle on post-hatch day 28 are shown in Table 2. Before heat stress (0 h, naïve), the basal expression of HSP108 mRNA expression in TM groups was significantly higher when compared with that of the control groups. Furthermore, after 1 h of heat stress, significant increases in mRNA expression of HSP108 occurred in all treatment groups and remained higher after 3 and 5 h of heat stress. Although there were reductions in the mRNA expression of HSP108 after 5 h, expression was still higher than in naïve chicks (0 h). Peak mRNA expression of HSP108 was observed in TM_1_, TM_4_ and control groups after 3 h of heat stress, whereas in TM_2_ and TM_3_, peak levels occurred after 1 h and 5 h, respectively. Before heat stress (0 h, naïve), basal mRNA expressions of HSP90 in all TM groups were significantly higher compared to the control group. Furthermore, after 1 h of heat stress, significant increases in mRNA expression of HSP90 were observed in all treatment groups and the elevated levels remained 3 h and 5 h of heat stress. The peak HSP90 mRNA level was observed in TM_1_, TM_4_ and control groups at 3 h of HS, whereas in the TM_2_ and TM_3_ groups, peak levels were observed after 1 h and 5 h of heat stress, respectively.

**Table 2:** The effect of TC (41 °C for 6 h; Post- Hatch Day 28) during 4 time terminals (at 0, 1, 3 and 5 h of TC) on relative mRNA expression of Hsp108, Hsp90, HSF-1 and HSF-2 in the Muscle of broiler chicks subjected to different TM protocols at ED 12–18 (*n* = 5)

Parameters	Groups	Treatments				
		Control	TM_1_	TM_2_	TM_3_	TM_4_
Hsp108	TN	1.00 ± 0.04^aw^	6.55 ± 0.70^bw^	2.15 ± 0.06^cw^	2.04 ± 0.14^cw^	2.89 ± 0.19^dw^
	TC 1	11.32 ± 0.50^ax^	8.11 ± 0.22^bx^	14.57 ± 0.13^cx^	11.37 ± 0.04^ax^	13.18 ± 0.16^dx^
	TC 3	15.74 ± 0.02^ay^	21.56 ± 0.06^by^	13.18 ± 0.25^cy^	13.77 ± 0.24^cy^	31.36 ± 0.62^dy^
	TC 5	14.81 ± 0.24^az^	7.96 ± 0.19^bz^	12.60 ± 0.05^cz^	14.39 ± 0.19^az^	9.15 ± 0.04^dz^
Hsp90	TN	1.00 ± 0.06^aw^	4.94 ± 0.36^bw^	2.35 ± 0.05^cw^	2.06 ± 0.04^cw^	2.83 ± 0.12^dw^
	TC 1	11.74 ± 0.60^ax^	7.68 ± 0.10^bx^	13.99 ± 0.07^cx^	11.18 ± 0.20^ax^	12.67 ± 0.20^dx^
	TC 3	17.35 ± 0.49^ay^	20.41 ± 0.59^by^	13.32 ± 0.29^cx^	13.52 ± 0.28^cy^	30.22 ± 0.92^dy^
	TC 5	15.47 ± 0.16^az^	7.54 ± 0.08^bx^	12.10 ± 0.14^cy^	13.22 ± 0.08^dy^	8.97 ± 0.25^ez^
HSF-1	TN	1.00 ± 0.08 ^aw^	1.16 ± 0.08^aw^	1.95 ± 0.07^bw^	1.02 ± 0.05^aw^	1.40 ± 0.08^cw^
	TC 1	2.07 ± 0.04^ax^	2.52 ± 0.12^bx^	2.23 ± 0.02^cx^	1.83 ± 0.01^dx^	1.94 ± 0.06^ax^
	TC 3	3.93 ± 0.11^ay^	2.24 ± 0.03^bx^	1.82 ± 0.07^cw^	1.23 ± 0.01^dw^	3.09 ± 0.03^ey^
	TC 5	1.67 ± 0.04^ax^	1.68 ± 0.04^ay^	2.83 ± 0.02^by^	1.99 ± 0.04^cx^	3.45 ± 0.05^dz^
HSF-2	TN	1.00 ± 0.04^aw^	3.79 ± 0.33^bw^	1.57 ± 0.04^cw^	1.40 ± 0.07^cw^	2.23 ± 0.07^dw^
	TC 1	2.26 ± 0.06^ax^	2.72 ± 0.10^bx^	3.08 ± 0.02^cx^	1.43 ± 0.01^dw^	3.33 ± 0.07^ex^
	TC 3	3.14 ± 0.04^ay^	2.17 ± 0.01^by^	1.61 ± 0.02^cw^	2.70 ± 0.02^dx^	5.72 ± 0.09^ey^
	TC 5	3.01 ± 0.07^ay^	5.27 ± 0.25^bz^	2.93 ± 0.03^ax^	3.18 ± 0.15^ay^	3.99 ± 0.06^cz^

*TC* Thermal challenged, *TM* thermal manipulation, *TN* Naїve (thermal-neutral). Control = 37.8°C; TM_1_ = Thermal manipulation at 38.5°C for 18 h; TM_2_ = Thermal manipulation at 39 °C for 18 h; TM_3_ = Thermal manipulation at 39.5°C for 18 h; TM_4_ = Thermal manipulation at 40 °C for 18 h

^a–e^within rows, means ± SD with different superscripts differ significantly (*P* < 0.05)

^w-z^Between naïve and TC chicks within a parameter, means ± SD with different superscripts differ significantly (*P* < 0.05)

#### mRNA expression of HSFs

Effects of TM and HS on mRNA expression of HSFs in the muscle on post-hatch day 28 are shown in Table 2. Before heat stress (0 h, naïve), the basal mRNA expression of HSF-1 in TM_2_ and TM_4_ groups was significantly higher compared to TM_1_, TM_3_ and control groups. However, after 1 h of heat stress, significant increases in mRNA expression of HSF-1 were reported in all treatment groups with the largest increase occurring in the TM1 group. The peak mRNA level of HSF-1 was observed after 1 h of heat stress in the TM1 group, whereas in TM2, TM3 and TM4, the peak mRNA level of HSF-1 was found after 5 h of heat stress. In contrast, in the control group, the peak mRNA level of HSF-1 was observed after 3 h of heat stress. Before heat stress (0 h, naïve), the basal mRNA expression of HSF-2 in TM groups was significantly higher compared to the control group. However, after 1 h of heat stress, significant increases in mRNA expression of HSF-2 were observed in all treatment groups, with the largest increase occurring in the TM_1_ and TM_4_ groups. The peak mRNA level of HSF-2 was observed 5 h after the beginning of heat stress in TM_1_ and TM_3_ groups, whereas in control and TM_4_ the peak mRNA level of HSF-2 was observed 3 h after the beginning of heat stress. In contrast, the peak mRNA level of HSF-2 in the TM_2_ was observed after 1 h of TM_4_.

## Discussion

The purpose of the current study was to investigate effects of thermal manipulation during broiler chicken embryogenesis on the basal and dynamics mRNA expression of HSP108, HSP90, HSF-1 and HSF-2 in the muscle during late embryogenesis, first week of age and during heat stress on post-hatch days 14 and 28. Our recently published report, involving similar thermal manipulations (38.5, 39, 39.5 or 40 °C daily for 18 h) during broiler chicken embryogenesis (ED 12–18), reported that these TM treatments had no effect on the body temperature at any stage of development (post-hatch days 1–35). However, during heat stress on post-hatch days 14 and 28, the body temperature of treated chicks was significantly lower than that of the controls [19]. It has been reported that, HSP108 is expressed constitutively in many chicken tissues and is induced by heat stress in primary cell cultures and chicken oviduct [25]. Previously, we reported that heat stress in thermally manipulated broiler chicken led to significant increases in the mRNA levels of HSP108 in heart, brain and muscle tissues on post-hatch days 14 and 28 [20]. However, in these experiments, the changes in the mRNA expression of HSP108 were only evaluated at a single-time-point (after 6 h of heat stress). This is the first study to report the dynamics of HSP108 mRNA expression during heat stress in TM treated chicken. TM resulted in significant increases in the basal mRNA expression of HSP108 on ED 18 and on post-hatch days 1, 5, 14 and 28. Furthermore, during heat stress on post-hatch days 14 and 28, a rapid induction of HSP108 was observed with the peak expression was observed in TM_4_ after 1 h from the beginning of heat stress while for the other TM groups (TM_1_, TM_2_ and TM_3_) the peak of expression was observed after 3 h from the beginning of heat stress. This indicates that TM has a short- and a long-term effect in the expression of HSP108 in the TM groups. The short-term effect is manifested by the alteration in basal expression of HSP108 in TM groups during embryogenesis and post-hatch days, whereas the long-term effect can be seen by alteration in the dynamics of mRNA expression of HSP108 during heat stress. After 5 h of heat stress, the mRNA expression of HSP108 is reduced in the TM groups compared to the controls. This indicates not only TM alters the basal and the dynamics mRNA expressions of HSP108 during heat stress, but also alters the kinetics of recovery of HSP108 after heat stress. HSP90, an essential molecular chaperone in eukaryotic cells, plays major roles in managing protein folding, protein degradation and activation of proteins involved in signal transduction and control of the cell cycle [26]. Furthermore, it has been reported that HSP90 has a dual involvement in signal transduction and cellular responses to heat stress [27]. It has been observed that, heat stress at post-hatch days 14 and 28 of age increased the mRNA levels of HSP90 significantly in heart, brain and muscle tissues whereas, the changes in the mRNA expression of HSP90 were evaluated at a single-time-point (after 6 h of heat stress) [23]. The current study reports for the first time the dynamics of HSP90 mRNA expression during heat stress in TM treated chicken. TM resulted in significant increases in the basal mRNA expression of HSP90 on ED 18 and on post-hatch days 1, 5, 14 and 28. During heat stress on post-hatch days 14, a rapid induction of HSP90 was observed. The peak expression of HSP90 was observed in TM_4_ after 1 h from the beginning of heat stress and for the other TM groups (TM_1_, TM_2_ and TM_3_) was observed after 3 h from the beginning of heat stress. Furthermore, during heat stress on post-hatch days 28, a rapid induction of HSP90 was observed. The peak expression of HSP90 was observed in TM_2_ after 1 h from the beginning of heat stress and for the other TM groups (TM_1_, TM_3_ and TM_4_) was observed after 3 h from the beginning of heat stress. This also indicates that TM has a short-term and a long-term effect in the expression of HSP90 in the treated groups. These results indicate that, TM had a long-lasting effect on HSP90 expression, with a rapid increase in HSP90 that could account for the improvement in thermotolerance acquisition and tissue stability in the face of hyperthermia in the TM groups. Heat shock factors (HSFs) are transcription factors that regulate the expression of heat shock proteins [10, 19, 21, 23, 28]. Four heat shock factors are known to regulate the expression of heat shock proteins: HSF-1, HSF-2, HSF-3, and HSF-4 [10, 19, 21, 23]. HSF1 is the master regulator of the heat shock genes [29]. Furthermore, HSF2 is crucial for development [29, 30] and also participates in HSF1-mediated HSP expression through formation of a heterocomplex with HSF1 [29]. In the current study, TM resulted in significant alterations in the mRNA expression of HSF-1 and HSF-2 in TM chicks at ED 12 and 18 and on post-hatch days 1, 5 and 7. Furthermore, during heat stress on post-hatch days 14 and 28, the mRNA expression of HSF-1 and HSF-2 were significantly higher than that of the control and naïve chicks. Interestingly, the increases in HSFs mRNA expression were coincided with the increases of HSPs mRNA expression. This indicates that TM during embryogenesis has short-term and long-term effects on the expression of HSFs in broiler chickens, which occur after cessation of TM. Furthermore, this alteration was associated with improved thermotolerance acquisition in the broiler chicken during heat stress.

## Conclusion

The results of this study indicate that thermal manipulation of broiler chicken eggs (38.5°C, 39 °C, 39.5°C and 40 °C for 18 h) daily during ED12–18 led to an improvement in thermotolerance acquisition in TM chicks during heat stress. This improvement could be attributed to the observation that TM not only altered the basal expression of HSP108, HSP90, HSF-1 and HSF-2 during late embryogenesis and the first week of life but also resulted in alterations in the dynamics of the mRNA expression of these HSPs and HSFs during heat stress. This suggested that TM during broiler chicken embryogenesis may improve thermotolerance acquisition in chickens raised in regions with high ambient temperatures.

## Abbreviations

ED: Embryonic days
HS: Heat stress
HSF: Heat Shock Factor
HSP: Heat shock protein
RH: Relative humidity
TC: Thermal challenged
TM: Thermal manipulation
